# Myasthenia gravis and acetylcholine receptor antibodies: A clinico immunological correlative study on South Indian patients

**DOI:** 10.4103/0972-2327.44560

**Published:** 2008

**Authors:** P. S. Bindu, M. Nirmala, S. A. Patil, A. B. Taly

**Affiliations:** Department of Neurology, National Institute of Mental Health and Neurosciences, Bangalore, India; 1 Department of Neuromicrobiology, National Institute of Mental Health and Neurosciences, Bangalore, India

**Keywords:** AChR antibodies, enzyme linked immunosorbent assay, myasthenia gravis, seronegative myasthenia Gravis

## Abstract

Acquired myasthenia Gravis (MG), a disorder of impaired neuromuscular transmission is recognized as an autoimmune disorder, with a majority of the patients having antibodies against acetylcholine receptor (AChR antibodies) in the serum.

**Aim and Objectives:**

To correlate the AChR antibody status with the clinical characteristics of patients with myasthenia gravis.

**Study Design:**

Retrospective and prospective study.

**Materials and Methods:**

This study was carried out in patients with definite MG, attending the Neurology services of the National Institute of Mental Health and Neurosciences, Bangalore, India, during the period 1999-2003. The AChR antibody status was determined using the direct and indirect enzyme linked immunosorbent assay (ELISA) technique.

**Results:**

There were 165 patients in this study (M : F :: 1.5 : 1). The overall seropositivity rate was 59.4%. Seropositive patients had higher age of onset and presentation, and more frequent occurrence of crises, both at presentation and at any time during the course. Other parameters, viz. gender of the patient, Osserman staging, thymic enlargement on CT and remission during follow-up did not differ between the two groups.

**Conclusion:**

This communication reports the result of AChR antibody assay in a large cohort of patients, using a simple diagnostic tool, namely direct and indirect ELISA technique. In addition, the characteristics of a large cohort of patients with seronegative myasthenia gravis are described.

## Introduction

Myasthenia gravis is the most common primary disorder of neuromuscular transmission. Acetylcholine receptor (AChR) antibodies are detected in the serum of more than 80-90% patients with generalized myasthenia gravis, about 50% with pure ocular myasthenia and rarely in healthy people.[1] There is a paucity of literature on the serological status of myasthenia gravis patients from India. In this study, an attempt is made to determine the AChR antibody positivity in a large cohort of patients with myasthenia gravis, using enzyme linked immunosorbent assay (ELISA) technique and its possible relation to the clinical status.

## Materials and Methods

This study included 165 patients diagnosed with definite myasthenia gravis, attending the Neurology services of the National Institute of Mental Health and Neuro Sciences, Bangalore, during 1999-2003. The diagnosis was based on characteristic history, bedside fatigability tests, positive neostigmine test, and repetitive nerve stimulation test. Patients with onset at birth (Congenital myasthenia) and family history of myasthenia gravis (Familial MG) were excluded from the analysis. Clinical examination and bedside fatigability tests were carried out using a predesigned protocol. Special emphasis was given to the duration of the illness, severity of the disease and treatment received. The severity of the disease was assessed using Osserman and Genkin's staging system[2] and the patients were divided into different clinical staging at the time of diagnosis. Dantec or Nihon Kohden digital EMG system was used for the repetitive nerve stimulation study. A train of 10 supramaximal stimuli, at a rate of 3/s was used for stimulation. The responses were recorded from both proximal and distal muscles. Decrement of more than 10% from the first to the fourth compound muscle action potential responses was considered as positive. Post exercise facilitation and post exercise exhaustion were studied after one minute of isometric exercise of the muscle being tested, whenever required. AChR antibody testing was done using both direct and indirect ELISA techniques[3,4] and the test was considered positive only when both the tests were positive.

## Results

In this study, the AChR antibody status of a large cohort of patients with myasthenia gravis was evaluated. Diagnosis of MG was established by typical clinical history (100%), fatigability tests (97.5%), positive neostigmine test (97.2%) and decrement of more than 10% on repetitive nerve stimulation test (87.8%). There were 165 patients with definite acquired myasthenia gravis as defined above. This included 98 males and 67 females patients (M : F :: 1.5 : 1). Their serological status in the different groups is shown in Table 1. Seropositive and seronegative groups were compared with regard to age at onset and presentation, gender, presence or absence of crisis at the time of presentation or at any time during the course of follow-up, presence of abnormal thymus on CT scan of the chest among individuals above the age of 15 years, history of remissions, and mortality [Table 2]. It was noted that seropositive patients had higher age of onset and presentation, and more frequent occurrence of crises, both at presentation and at any time during the course. Other parameters, viz.gender of the patient, Osserman staging, thymic enlargement on CT and remission during follow-up did not differ between the two groups.

**Table 1:** Serological status of patients with myasthenia gravis

Group	n	Seropositive	Percentage
Myasthenia gravis	165	98	59.4
Ocular	39	20	51.28
II a	71	40	56.33
II b	34	22	64.70
III	18	13	72.20
IV	03	03	100

**Table 2:** Comparison of various parameters between anti-acetylcholine receptor antibody positive and negative patients with Myasthenia gravis (*n* - 165)

Parameter	Seropositive (n = 98)	Seronegative (n = 67)	*P* value
Mean age at presentation	37.8 ± 16.5	30.7 ± 18.5	0.01
Age at onset (years)	35.2 ± 21.65	28.7 ± 19.27	0.05
Gender ratio (M:F:: 98:67)	64 : 34	34 : 33	0.06*
Stage			
Ocular V/s Others (39 :126)	20 :78	19 : 48	0.24*
Mild V/s Severe (110 : 45)	60 : 38	50 : 17	0.07*
Crisis at presentation (n = 20)	17	3	0.01
Crisis at any time (n = 41)	30	11	0.05
Thymic enlargement on chest CT**	41	16	0.21*
Present (57): Absent (67)			
Remission (n = 28)	16 : 82	12 : 55	0.79*
Present (28): Absent (137)			
Deaths (n = 2)	96 : 2	67 : 0	0.35*
Present (2): Absent (163)			

Mild = Stage I and IIA

* Not significant

** Chest CT done in 124, Severe = IIB, III, IV Others = IIA, IIB, III, IV

## Discussion

In this study, we evaluated the AChR antibody status in a large cohort of patients with myasthenia gravis. Studies on the AChR status in myasthenia gravis, using ELISA, are few. The present study thus becomes relevant, particularly in Indian context.

Compared to earlier studies on myasthenia gravis this study shows a significant male preponderance, with a male-female ratio of 1.5 : 1. This is in commensuration with an another study from India[5] and a previous study from our institute.[6] This perhaps reflects the larger number of men seeking medical consultation in India, as our hospital statistics also shows a M:F ratio of 3:2.

The sero prevalence of AchR antibody in different series was varied, being in the 67-93% range and these antibodies are virtually absent in normal controls or in patients with other neurological or immunological diseases .[1,7] Jailkhani *et al*. used direct and indirect methods of ELISA and reported seropositivity in 90% of the patients of myasthenia gravis.[3] The overall antibody positivity rate observed in the present study is 60%. Compared to other studies, the positivity rate is low and may reflect a rather low sensitivity of ELISA used in this study.

There was antibody positivity in 51.28% of the patients with ocular myasthenia. While these values are lower than patients with generalized MG, the difference was not statistically significant. Patients with ocular MG in general have lower AChR antibody levels ranging from 40-75%.[8,9] They have the lowest mean antibody titer when measured against human limb muscle receptors, and in about 25% cases, the values are in the control range.[9] However, it has been noted that their sera react well with ocular muscle receptors.[7] This observation indicates that the antigenic differences between limb and ocular muscles may be responsible for the low positivity in ocular myasthenia. This also raises the possibility of the nature of the antibodies in ocular MG being different.

The role of thymus in the pathogenesis of myasthenia gravis is well-known. High antibody titers have been reported in patients with thymoma in MG.[1,7] In a previous study, it was noted that chest CT had a sensitivity of 73.1% and specificity of 75% in detecting thymic abnormalities.[10] In the present study, an abnormal thymus on CT did not predict the serological status.

Approximately, 12-17% of patients with generalized MG lack demonstrable serum AChR antibodies, and they are referred to as the seronegative group.[11–14] Attempts have been made to characterize this subgroup. Soliven *et al*. reported that there was no difference in the age of onset, gender, duration of symptoms or frequency of crises between the seropositive and seronegative patients.[13] None of the patients in the seronegative group had thymic enlargement on CT, in contrast to seropositive group. But this difference was not statistically significant. Vincent *et al*. noted that seronegative patients tended to have shorter duration or symptoms restricted to ocular muscles.[11] Sanders *et al*. observed that seronegative patients were more likely to be males and have milder disease, ocular myasthenia and fewer thymomas.[14] Lindstrom *et al*. could not find any consistent similarity among seronegative patients.[1] In the present study, it was noted that seropositive patients were older at presentation, had male preponderance and had more frequent occurrence of crises, both at presentation and at any time during the course of illness.

The newly discovered autoantibodies to muscle specific kinase (MuSK Antibodies) in the previously seronegative patients may help to understand the pathogenesis of seronegative patients. MuSK antibodies are reported to be present in two thirds of the AChR antibody negative patients.[15] In our series, the MuSK antibody status could not be assessed and so a comparison between the two groups could not be made.

In conclusion, this communication reports the result of AChR antibody assay in a large cohort of patients, using the direct and indirect ELISA technique. This brings about the usefulness of this simple diagnostic tool in the evaluation of patients with myasthenia. In addition, the characteristics of patients with seronegative myasthenia gravis have been described.
